# Paternal Low-Level Mosaicism-Caused *SATB2*-Associated Syndrome

**DOI:** 10.3389/fgene.2019.00630

**Published:** 2019-07-02

**Authors:** Yeqing Qian, Jiao Liu, Yanmei Yang, Min Chen, Chunlei Jin, Penglong Chen, Yongliang Lei, Hangyi Pan, Minyue Dong

**Affiliations:** ^1^Women’s Hospital, School of Medicine Zhejiang University, Hangzhou, China; ^2^Key Laboratory of Reproductive Genetics (Zhejiang University), Ministry of Education, Hangzhou, China; ^3^Prenatal Diagnosis Center, Lishui Maternity and Child Health Care Hospital, Lishui, China

**Keywords:** *SATB2*-associated syndrome, chromosome microarray analysis, mosaicism, droplet digital PCR, gap-PCR

## Abstract

Mutations of *SATB2* (OMIM#608148) gene at 2q33.1 have been associated with the autosomal dominant *SATB2*-associated syndrome (SAS), which is still short of comprehensive diagnosis technologies for small deletions and low-level mosaicism. In this Chinese Han family, single nucleotide polymorphism array identified a 4.9-kb deletion in the *SATB2* gene in two consecutive siblings exhibiting obvious developmental delay and dental abnormalities but failed to find so in their parents. Prenatal diagnosis revealed that their third child carried the same deletion in *SATB2* and the pregnancy was terminated. To determine the genetic causes behind the inheritance of *SATB2* deletion, gap-PCR was performed on peripheral blood-derived genomic DNA of the family and semen-derived DNA from the father. Gap-PCR that revealed the deletions in the two affected siblings were inherited from the father, while the less intense mutant band indicated the mosaicism of this mutation in the father. The deletion was 3,013 bp in size, spanning from chr2: 200,191,313-200,194,324 (hg19), and covering the entire exon 9 and part of intron 8 and 9 sequences. Droplet digital PCR demonstrated mosaicism percentage of 13.2% and 16.7% in peripheral blood-derived genomic DNA and semen-derived DNA of the father, respectively. Hereby, we describe a family of special AT-rich sequence-binding protein 2-associated syndrome caused by paternal low-level mosaicism and provide effective diagnostic technologies for intragenic deletions.

## Introduction

Special AT-rich sequence-binding protein 2 (SATB2) encodes a nuclear matrix DNA-binding protein that is involved in transcription regulation and chromatin remodeling. The *SATB2* gene spans 195.6 kb of genomic DNA (chr2:200,134,223-200,329,831, hg19) and is located at chromosome 2q33.1. SATB2 protein is composed of 733 amino acids, weighing approximately 82.5 kDa. The functional domains of SATB2 consist of two CUT domains (CUT1 and CUT2) that bind to the matrix attachment region and a homeodomain at the C-terminus (Fitzpatrick et al., 2003). All these domains are highly conserved across the vertebrate taxa (Sheehan-Rooney et al., 2010).

The *SATB2*-associated syndrome (SAS), which is a relatively newly described syndrome, is an autosomal dominant disorder also caused by alterations in the *SATB2* gene (Docker et al., 2014). SAS is characterized by neurodevelopmental disabilities (intellectual disability and absent or severely impaired speech), behavioral abnormalities (hyperactivity, sleeping difficulties, autistic features, obsessive tendencies, and/or aggressiveness), craniofacial anomalies (cleft palate or high-arched palate, dental abnormalities), and skeletal anomalies (tibial bowing, osteomalacia, osteopenia, or osteoporosis) (Docker et al., 2014; Bengani et al., 2017; Zarate and Fish, 2017; Zarate et al., 2017; Zarate et al., 2018b).The major features of SAS can be summarized by the acronym S.A.T.B.2: S, severe speech anomalies; A, abnormalities of the palate; T, teeth anomalies; B, behavioral issues with or without bone or brain MRI anomalies; and age of onset before 2 years of age (Docker et al., 2014). Dr. Yuri Zarate, one of the most famous experts in the SAS area, gave recommended diagnostic evaluation and health surveillance for SAS at the website www.satb2gene.com.

In this study, we report the first occurrence of phenotypically normal father with low-level mosaicism of intragenic deletion in the *SATB2* gene, which was identified in his two children with SAS. This deletion was initially hinted by chromosomal microarray (CMA) in affected children but failed to diagnose in their father, while mosaicism in father was ascertained by gap-PCR, and the percentages of mosaicism in both the peripheral blood and semen were determined by droplet digital PCR (ddPCR). Our study provided a novel diagnostic method for those intragenic deletions in families having more than two consecutive births carrying similar abnormalities.

## Materials and Methods

### Clinical Report

The 33-year-old pregnant woman was at 16 weeks of gestation. Her husband, 38 years old, has a normal phenotype as his wife does. No family history of mental or behavioral abnormalities or any other intellectual disability were reported. They came to our hospital for prenatal diagnosis because their two children had severe developmental delay. The husband’s mother was diagnosed with esophageal cancer, and she died of multiple tumors at the age of 50.

The proband, male, 11 years old, weighed 28.6 kg (10th percentile), 136.6 cm tall (10th percentile), and had a low bone density (1st percentile). Main clinical manifestations consist of intellectual disability, no speech, high-arched palate, dental abnormalities (crowding and abnormal shape), small jaw, short tongue strap (surgically corrected), hypotonia, poor appetite, salivation, behavior abnormality (aggressiveness), and hydrocele. The head MRI examination showed brain demyelinating disease at the age of 3. He started walking at 4 years of age.

His sister, female, 8 years old, weighed 19.0 kg (3th percentile), 115.2 cm tall (3th percentile), and low bone density (11th percentile). Main clinical manifestations consist of intellectual disability, severely impaired speech (can only call dad and mom), high-arched palate, dental abnormalities (crowding and abnormal shape), small jaw, short tongue strap (received surgery), hypotonia, poor appetite, and salivation. She started walking at the age of 2. She started showing epilepsy since 6 years of age, even though her head MRI showed normal results at the age of 6. Her intellectual abilities were below an average 7 years old.

The karyotype analysis of G-banding in the peripheral blood of two siblings and parents showed a standard chromosomal pattern (data not shown). CMA result of both the proband and his sister using CytoScan™ HD whole genome SNP array (Affymetrix, USA) showed two copy number variations (CNVs): arr[hg19] 2q33.1(200,192,328-200,197,269)x1,2q35(218,105,663-218,816,675)x3. They have a 4.9-kb deletion in the 2q33.1 region of chromosome 2 (**Figure S1**). The deletion spans the exon 9 of the *SATB2* (OMIM: 608148) gene. They also have a 711-kb duplication (arr[hg19] 2q35(218,105,663-218,810,908)x3) in the 2q35 region, which contains *DIRC3* (OMIM: 608262) and *TNS1* (OMIM: 600076) genes. There has been no clear disease-related report on the duplication of this fragment or on these two genes. The mother’s CMA result: arr[hg19] 5q31.3q33.1 (141,383,108-151,756,016)x2 hmz. The father’s CMA results: arr[hg19] 2q35 (218,105,663-218,810,908)x3. The couple did not carry the 4.9-kb deletion at 2q33.1, while the 711-kb duplication at 2q35 of the children should have been inherited from their father. However, the possibility of a low-level somatic or gonadal mosaicism was not excluded. Meanwhile, the exact breakpoints were yet to be determined.

The pregnant woman received amniocentesis at 19 weeks of gestation. The karyotype of the fetus was normal, but CMA result was the same as those of the affected children. In addition, the B-ultrasound showed that the fetus had ventricular septal defect. After genetic counseling and through consideration, the couple chose to terminate the pregnancy at 23 weeks of gestation.

This study was carried out in accordance with the recommendations of the Ethics Committee of Women’s Hospital, School of Medicine Zhejiang University. All subjects gave written informed consent in accordance with the Declaration of Helsinki, and written informed consent was also obtained from the guardians of the two affected siblings for the publication of this case report. The protocol was approved by the Ethics Committee of Women’s Hospital, School of Medicine Zhejiang University.

### Peripheral Blood DNA Extraction

**Figure 1:**
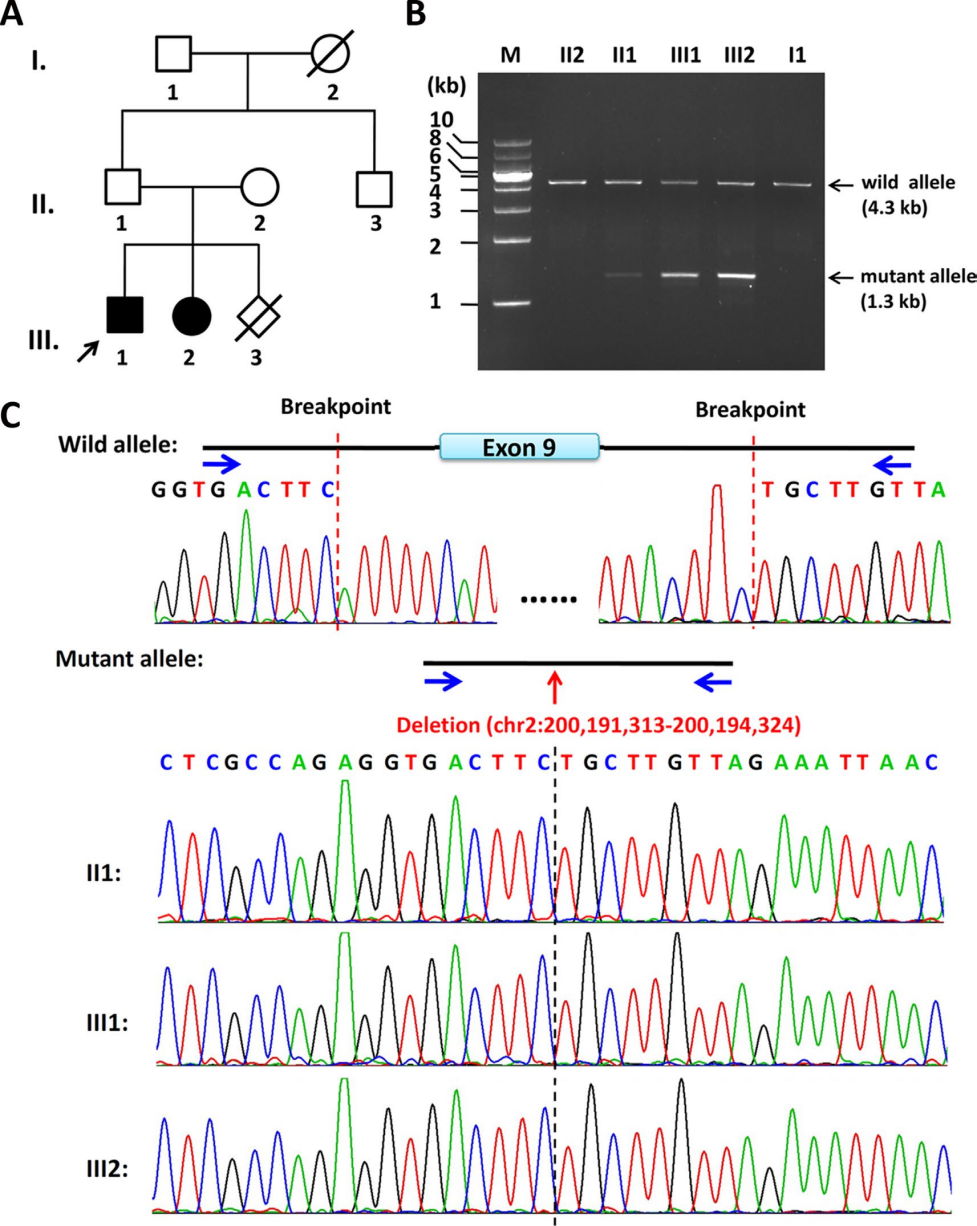
Pedigree of the family and the characterization of the deletion breakpoints by gap-PCR. **(A)** Pedigree of this family. The arrow refers to the proband. The solid square (male) and circle (female) represent the two affected siblings; **(B)** Gap-PCR of family members using the peripheral blood-derived genomic DNA and primer sets (4334-F/R). Lanes 1–7: Marker, II2, II1, III1, III2, II3, and I1. The expected *SATB2* wild allele band of ∼4.3 kb is seen in members of II2, II1, III1, III2, II3, and I1. A lower band, consistent with the size of mutant allele, is seen in II1, III1, and III2 but not in II2 or I1. Notice that the father (II1) has a much less intense band of about 1.3 kb in size, which is the same size as his two children (III1 and III2). **(C)** Sequencing results of the ∼4.3-kb band and 1.3-kb band. The intragenic deletion border is chr2: 200,191,313-200,194,324 (hg19), which includes the entire exon 9 and its franking intronic sequences of the *SATB2* gene. Blue arrows refer to gap-PCR primer set (4334-F/R) used to detect the breakpoints of the 3kb deletion.

Peripheral blood samples were collected from the family members including the husband’s father (I1), the couple (II1 and II2), and their two children bearing SAS (III1 and III2) (**Figure 1A**). Genomic DNA was extracted from peripheral blood samples using the QIAGEN spin columns on a QIACube (QIAGEN GmbH) according to the manufacturer’s instructions. All DNA samples were dissolved in water and stored at −20°C.

### Gap-PCR and Primer Walking

Gap-PCR was chosen for locating exact breakpoints of the deletion. Three sets of primers (Gap2500-F/R, Gap1000-F/R, Gap500-F/R, **Table 1**) were designed according to the CMA results. Gap2500-F/R primer set worked well using TaKaRa LA Taq™ (TaKaRa Bio Inc.) and two-step procedure. The procedure of the PCR was as follows: 94°C for 1 min followed by 30 cycles at 98°C for 10 s, 68°C for 8 min, and a final extension step at 72°C for 10 min. Based on this result, another four primer sets (6666-F/R, 5335-F/R, 4334-F/R, 3442-F/R, **Table 1**) were designed to amplify more suitable size of fragments for sequencing. The procedure of the PCR was as follows: 94°C for 1 min followed by 30 cycles at 98°C for 10 s, 68°C for 4 min, and a final extension step at 72°C for 10 min.

**Table 1:** Gap-PCR primers.

Primer	Direction	Sequence (5’-3’)	Product length (bp)
Gap2500-F	F	ATCAAATTGTCATTGTTGTGCC	9,376
Gap2500-R	R	CCATCTAAACCTCAGTTCCCTC	
Gap1000-F	F	ATAATACCTTCTCCTTCCCATC	6,575
Gap1000-R	R	TTAACAACTTGCCCAACTTACT	
Gap500-F	F	GTTAAAGGCCCATCAAAGGTAA	5,667
Gap500-R	R	GTTCGAGACCAGCATGGACAAC	
6666-F	F	GATAATCAATGGGAGATAATGG	6,666
6666-R	R	CCTTGTAAGTCAGTCTGGCACT	
5335-F	F	TCTTTCCCATAATAAACTCCAC	5,335
5335-R	R	TCTAAACCTCAGTTCCCTCATC	
4334-F	F	AAATGTCTTTGGCATCTGTTC	4,334
4334-R	R	CCATCTAAACCTCAGTTCCCT	
3442-F	F	ACAATTCTTCCCAAGTGCCTAC	3,443
3442-R	R	CATCTAAACCTCAGTTCCCTCA	

### Sperm Preparation and Genomic DNA Extraction

The semen sample of the father was obtained by masturbation following 5 days of sexual abstinence and then was allowed to liquefy for 60 min at 37°C before processing. Semen sample was diluted 1:2 in Earle’s solution, centrifuged at 500×g for 5 min. Then sperm pellets were washed twice with Earle’s solution, and the final pellets were preserved immediately in liquid nitrogen for DNA extraction. Pellets were homogenized, and genomic DNA was extracted using the QIAGEN spin columns on a QIACube (QIAGEN GmbH) according to the manufacturer’s instructions.

### Real-Time Quantitative PCR

**Figure 2:**
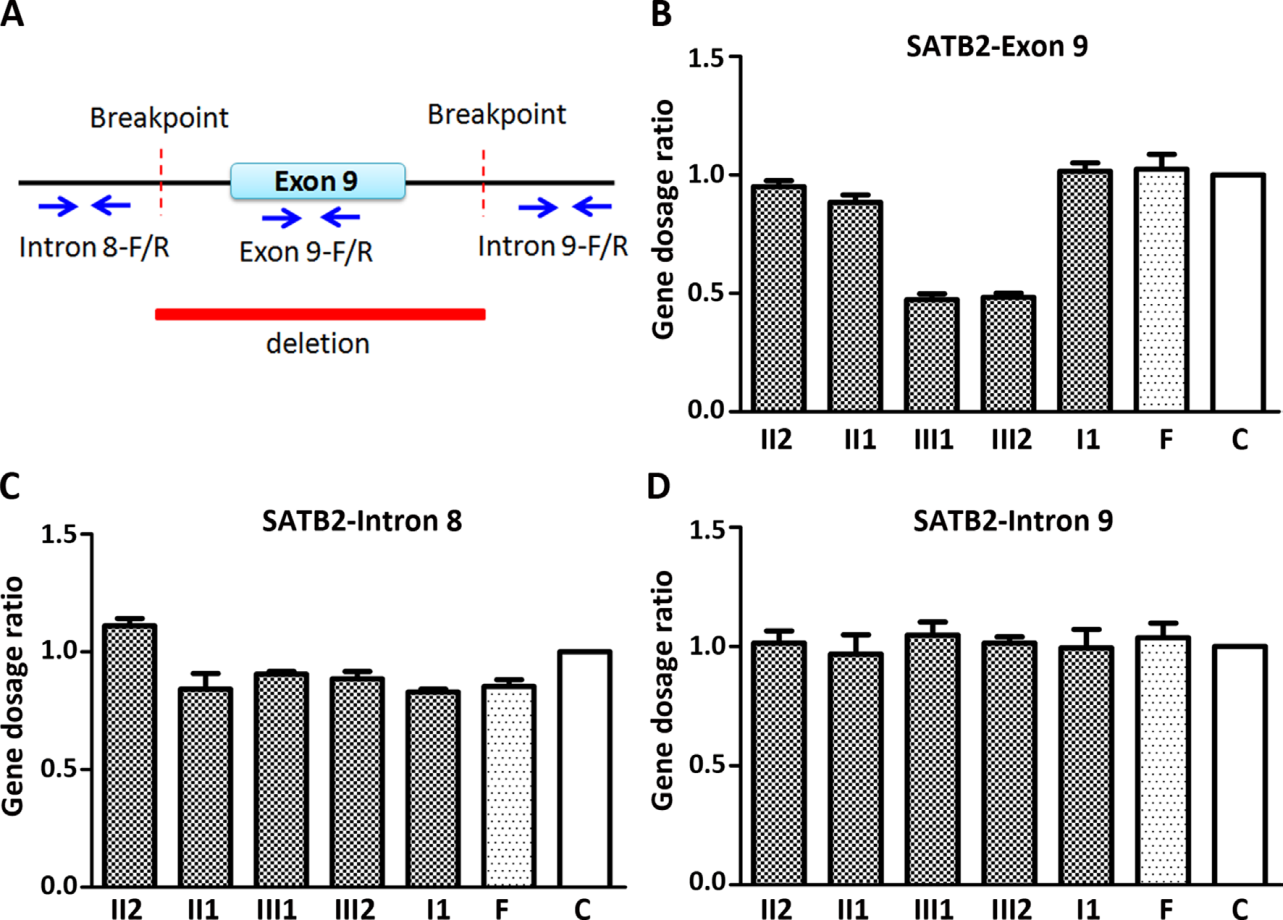
Quantitative PCR (qPCR) of family members using the peripheral blood-derived genomic DNA. **(A)** Schematic diagram of location of qPCR primers. Blue arrows refer to qPCR primers; **(B)** Comparison of gene dosage ratios for the family members using the SATB2-exon 9 primers; **(C)** Comparison of gene dosage ratios for the family members using the SATB2-intron 8 primers; **(D)** Comparison of gene dosage ratios for the family members using the SATB2-intron 9 primers. F, normal female; C, control (normal male).

Three pairs of primers (**Figure 2A**) were designed to detect potential deletions in this family, including two primer sets outside the deletion region and one primer set inside the deletion. The design of primers is in accordance with the following standard: the length of the amplified product is less than 300 bp, and annealing temperature is close to 60°C. The three pairs of primers were designed by Primer-BLAST software (http://www.ncbi.nlm.nih.gov/tools/primer-blast/) (see **Table 2**). Specificity and effectiveness have been examined in preliminary experiment. Furthermore, β-globin was chosen as reference gene (forward primer: 5’-ACACAACTGTGTTCACTAGC-3’; reverse primer: 5’-CAACTTCATCCACGTTCACC-3’). The reaction was performed on a LightCycler 480 II real-time PCR system (Roche) in a final volume of 20 µl containing 10 µl of SYBR Premix Ex Taq Polymerase (TaKaRa), 0.4 µl each of the forward and reverse primers (10 µmol/l), and 2 µl of the genomic DNA (20 ng). Each sample was tested in triplets. The procedure of the PCR was as follows: 95°C for 10 s, followed by 40 cycles at 95°C for 5 s, 58°C for 30 s, and a final extension step at 72°C for 10 min. The dosage ratio of the tested samples was calculated by the ΔΔCt method for each test sample according to Livak and Schmittgen (2001).

**Table 2:** Quantitative PCR (qPCR) primers.

Primer	Direction	Sequence (5’-3’)	Product length (bp)
Intron 8-F	F	ATGGAAATAGACCTGCACCTAC	186
Intron 8-R	R	TGACTCACCCAAATAGAAAGAT	
Exon 9-F	F	GATTCTGCGTAAGGAAGAAGACC	158
Exon 9-R	R	AGACCATGCTCACATTGGGATT	
Intron 9-F	F	ACCTAATAGCTTTCAGTGCCAGAC	130
Intron 9-R	R	TTCCCTGCTACACCTATCCCTA	

### Droplet Digital PCR

The somatic and gonadal mosaicism levels of the father were estimated using the Pilot Droplet Digital PCR system [Pilot Gene Technologies (Hangzhou) Co., Ltd. China]. The primer set SATB2Del-F: CAGAAAGTATCTGGGCCTATCAT/SATB2Del-R: GTACAAAAGAGCTGAAAACAAATACA, which was located on each side of the breakpoint, were used along with the 6-carboxyl-x-rhodamine (ROX)-labeled fluorescence probe SATB2Del-P: ROX-AGGTGACTTCTGCTTGTT-MGB, which spanned the deletion region to detect the copy number of the mutation. Another primer–probe set PARP2-F: GCGGAGGGAAGCTCATCAGTG/PARP2-R: CCCTAGTCTCAGACCTTCCCAA/PARP2-P: 6-FAM-ACATGGGAGTGGAGTGACAGG-BHQ1 was employed to detect the single copy reference gene Poly (ADP-ribose) polymerase 2 (PARP2) in this study. The mosaic ratio was calculated as the copy number of mutation/the copy number of the reference gene.

The droplet generation, PCR amplification, and chip scanning were performed according to the manufacturer’s instructions. Briefly, 20 µl of PCR mixture containing 1× PCR buffer, 1 µl each of the genomic DNA, 150 nM of 6-FAM-labeled probe, 600 nM of ROX-labeled probe, and 600 nM of each primer were loaded into Pilot droplet chips. Droplets were automatically generated in the Pilot droplet generator. Chips were then transferred into the Pilot iThermal1.0, and samples were amplified using the following cycling settings: initial denaturation at 95°C for 10 min, followed by 40 cycles of 95°C for 30 s, and 55°C for 60 s. Post-PCR chips were scanned using Pilot iScanner5 chip scanner, and results were analyzed using the GenePMSv1.1 software.

## Results

### The Deletion Is of Paternal Origin and Includes Part of Intron 8, the Entire Exon 9, and Part of Intron 9 of *SATB2*

By the clinical diagnosis and CMA, we narrowed down the target of interest to *SATB2*. To make sure the exact breakpoints of *SATB2* mutation, three primer sets were designed outside the region indicated by the CMA result. One of the three gap-PCR primer sets (Gap2500-F/R) worked well, and gel electrophoresis results showed both father, and the two siblings share two amplified bands (**Figure S2**). The larger band is about 10 kb in size, and the smaller band is about 7 kb in size. The 7-kb band of the father is less intense than that of the two siblings. Based on this result, another four primer sets were designed to amplify fragments with proper lengths for Sanger sequencing. Gap-PCR products using primers (4334-F/R) of all the family members shared the 4.3-kb band, while gap-PCR products of II2 (father), III1 (the proband), and III2 (the sister) have another smaller band (∼1.3kb) (**Figure 1B**). The Sanger sequencing result of the smaller band of the father and the two siblings revealed that the breakpoints were located at chr2: 200,191,313-200,194,324 (hg19) when blasted with genomic sequence (NM_001172517). The deleted region includes part of intron 8, the entire exon 9, and part of intron 9 (**Figure 1C**). Given that the father has no phenotype and the father carries a smaller band of lower intensity comparing with those of the two siblings, the father might have a low level of mosaicism.

### Deletion Is Present in Peripheral Blood and Semen of the Father

In order to know the likelihood of recurrence of the deletion in the next pregnancy of the mother, gap-PCR using primers 4334-F/R was performed on semen-derived DNA of the father. Gel electrophoresis results that showed both amplified products of semen-derived DNA and peripheral-blood DNA of the father share two amplified bands with same sizes (**Figure S3**). We compared the smaller mutant band between the amplified products of both DNA samples by Sanger sequencing, and the sequencing results showed they were all deleted mutant (data not shown). We found that the father has not only somatic deletion but also germline deletion (since it is found in the semen); hence, the risk of the fetus carrying the same deletion in the next pregnancy still exists.

### The Two Siblings Had One Copy in the Deletion Region by Quantitative PCR

Since CMA and gap-PCR are having conflicting results regarding the deletion of father, quantitative PCR (qPCR) was carried out using three primer sets (**Figure 2A**) in the family members to determine which test result is correct. Because of the deletion region including part of intron 8, the entire exon 9, and part of intron 9, we examined the exon 9 (representative of deletion region) and intron 8 and 9 (representative of non-deletion region) of the *SATB2* gene by the qPCR. The level of exon 9 in the two siblings is about half of the normal level in other family members (**Figure 2B**). The result of intron 8 and 9 showed no obvious differences in the family members (**Figure 2C and D**). The above results confirmed that both CMA and gap-PCR were right about the level of deletion in the two siblings but failed to justify the gap-PCR results about the father’s deletion level. Further confirmation on father’s deletion level is required.

### The Father Was Low-Level Somatic and Gonadal Mosaicism in the *SATB2* Gene

**Figure 3:**
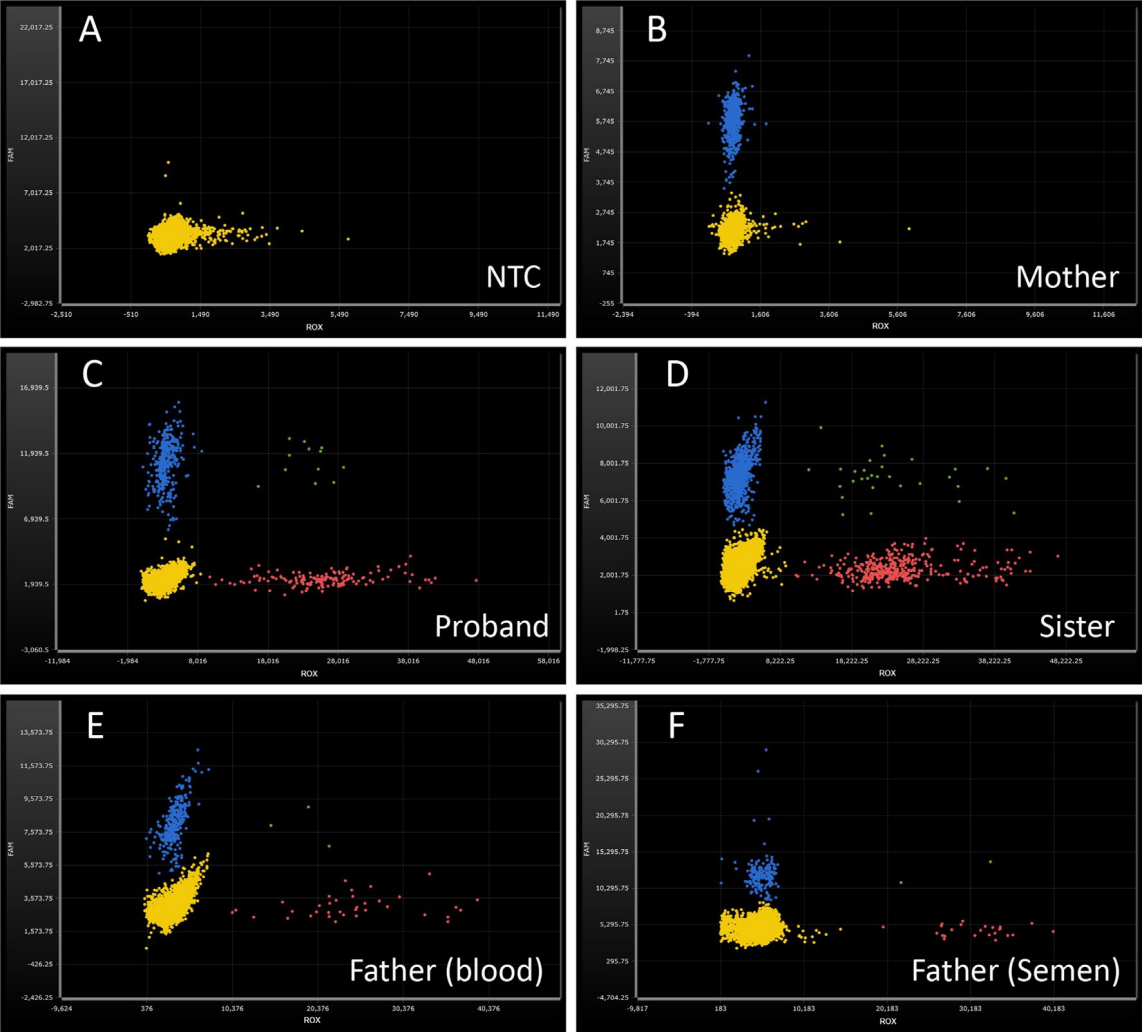
Droplet digital PCR analysis for the mutant frequencies of *SATB2* gene. ROX-labeled probe detects the delete mutation of *SATB2* gene (red dots). 6-FAM-labeled probe detects the reference gene PARP2 (blue dots). Green dots, ROX and 6-FAM double positive. Yellow dots, no amplification. **(A)** No-template control, NTC; no amplification on both fluorescence channels. **(B)** wild type, DNA from the proband’s mother; normal amplification on FAM channel, while no amplification on ROX channel. **(C, D)** heterozygosity, DNA from the proband and his sister, respectively; the ratios of ROX/FAM were close to 0.50. **(E, F)** mosaicism, DNA from the proband’s peripheral blood and semen of the father; the ratios of ROX/FAM were 13.16% and 16.68%, respectively.

In order to check the level of somatic and gonadal mosaicism of the *SATB2* gene in the father, ddPCR was used to estimate the frequencies of the mutant allele in this study. As shown in **Figure 3** and **Table 3**, compared with the frequencies of no-template control (**Figure 3A**), the mutant frequencies of the proband and his sister were 52.88% (**Figure 3C**) and 52.11% (**Figure 3D**), respectively, indicating heterozygosity on the detected site for both of them, while their mother has zero mutant frequency (**Figure 3B**), signifying wild type on the detected site. Two DNA samples from peripheral blood and semen of the proband’s father were analyzed by ddPCR in this study, which showed the mutant frequencies of 13.16% (**Figure 3E**) and 16.68% (**Figure 3F**), respectively. Above results confirmed the low-level mosaicism in the *SATB2* gene of the father. Gap-PCR and ddPCR together provide an effective diagnostic technique for this intragenic deletion.

**Table 3:** Mutant frequencies of *SATB2* gene detected by Droplet Digital PCR.

Subject	PARP2 concentration (FAM) copies/µl	SATB2 concentration (ROX) copies/µl	Mutant frequency (ROX/FAM, %)
Father (blood)	48.080	6.326	13.16
Father (semen)	21.978	3.667	16.68
Mother	147.06	0	0
Proband	65.415	34.595	52.88
Sister	62.813	32.733	52.11

## Discussion

Glass syndrome or 2q33.1 microdeletion syndrome (OMIM#612313) was first described by Glass et al. (1989) in a 16-year-old male with cytogenetically visible deletion of 2q32.2-q33.1, and he had severe intellectual disability, cleft palate, short stature, and craniofacial dysmorphism (Glass et al., 1989). This syndrome has a large deletion of 2q33.1 that includes the disease-causing gene *SATB2* and other genes. To reduce confusion and unify the nomenclature, a new term SATB2-associated syndrome (SAS) emerged. To date, intragenic deletions and duplications, point mutations involving *SATB2* gene have been reported as pathogenic SAS mutations in the literature (Zarate and Fish, 2017; Zarate et al., 2017; Zarate et al., 2018a).

To our knowledge, deletions have been reported in 22 patients thus far and ranged from 10 to 317 kb in size and with only *SATB2* included in the region (Rosenfeld et al., 2009; Balasubramanian et al., 2011; Zarate et al., 2018a; Zarate et al., 2019). Herein, the 3-kb intragenic deletion in the family of this study might be the smallest reported deletion in the *SATB2* gene thus far, and it is also covered within the 10-kb deletion reported in case ID 49 by Zarate et al. (Zarate et al., 2018a). ID 49 was an 18-year-old male carrying a 10-kb deletion (chr2:200,190,560-200,200,832, hg19) including the entire exon 9 and part of intron 8 and 9 sequences within the *SATB2* gene. He had growth retardation, cleft palate, feeding difficulties during infancy, sialorrhea, and abnormal MRI, and he started walking at the age of 12. We note that cleft palate and feeding difficulties during infancy were absent in our case, indicating that these characteristics may be variable in individuals affected by heterozygous deletion of *SATB2* exon 9. Sequence of exon 9 (coding 393–461 amino acids) covers C-terminal part of CUT1 domain (355–434). Among all SAS families with trio analysis by whole-exome sequencing or panels, Zarate and colleagues found that the most frequent pathogenic variant were missense mutations, and most of these variants localized to the CUT1 domain (Zarate et al., 2018a). That might suggest the importance of CUT1 (including exon 8 and 9) for the function of SATB2 protein. On the other hand, Bengani et al. constructed several mutant SATB2 plasmids and then transfected to Hela and human fibroblast cell lines. They found that cells with p.Arg389Cys change in CUT1 domain displayed more diffuse patterns of nuclear localization than the wild type, and the authors proposed that CUT1 domain may be required to initiate interaction with chromatin or matrix (Bengani et al., 2017). Therefore, considering the significance of CUT1 domain, exon 9 may be crucial to SAS symptoms.

Of the SAS-affected families reported thus far with trio analysis, two instances of mosaicism have now been documented in the literature. One case was presumed to be gonadal mosaicism because two affected siblings had the same mutation (c.582-2A>G), but none was detected in the leukocyte DNA of either parent (Bengani et al., 2017). Another case was also suspected to be gonadal mosaicism for the two affected siblings share an identical 2.9-Mb micro-duplication that was not present in either parent (Bengani et al., 2017; Zarate et al., 2018a). In the present study, the father with normal phenotype in this family was proved to have a low-level somatic and gonadal mosaicism rather than both siblings carrying *de novo* mutations in the *SATB2* gene. The deletion in our case failed to detect due to low level of resolution in this specific region by SNP array (**Figure S1**); more details were only revealed with the help of gap-PCR and ddPCR. We recommend paternal semen sample testing as the first step because the acquisition of semen sample is easy and safe. If the results are negative, mutation from maternal germline origin could not be ruled out.

The real-time qPCR is another common method to determine copy number of target genes in addition to CMA; however, it does have several limitations such as failure to detect low-level mosaicism variations. This limitation can be overcome by ddPCR due to its ability to precisely quantify mosaic genomic copy number variations when compared with qPCR (Fujiki et al., 2016; Taylor et al., 2017; Zhou et al., 2018). The proband’s father was mosaic in *SATB2*, estimated to be 13.16% in the peripheral blood and 16.68% in the semen by ddPCR. With mosaicism detected in semen as well, recurrence risks are higher compared with those phenotypes occurring from *de novo* variants. It is difficult to estimate an accurate recurrence risk if the percent mosaicism in the gonad is unknown.

Our study further validates the need to perform trio analysis if two phenotypically normal parents had two or more consecutive pregnancies with similar SAS phenotypes. In terms of parental testing, CMA and traditional qPCR are not sensitive enough as exemplified in our study. Gap-PCR and ddPCR are more sensitive for detecting low-level mosaicism, and ddPCR may further be used for estimation of mutant frequencies, if needed. The different results have greatly different reproductive recurrence risks, that is, apparently, *de novo SATB2* pathogenic variants confer very low recurrence risk versus paternal mosaicism that confers up to a 50% recurrence risk.

In conclusion, CMA is recommended as the first-tier test followed by gap-PCR and ddPCR if a similar situation is arising. We highlighted the importance of detailed genetic testing, testing method, and counseling for cases of somatic or gonadal mosaicism in an unaffected parent of children with SAS syndrome. Due to inconclusiveness of CMA results alone, we suspect that this inheritance might be underreported and the results would have a direct impact on reproductive planning and prenatal diagnosis.

## Data Availability Statement

All datasets are included in the manuscript or supplementary files.

## Ethics Statement

This study was carried out in accordance with the recommendations of Ethics Committee of Women’s Hospital, School of Medicine Zhejiang University, with written informed consent from all subjects. All subjects gave written informed consent in accordance with the Declaration of Helsinki. The protocol was approved by the Ethics Committee of Women’s Hospital, School of Medicine Zhejiang University.

## Author Contributions

MD, YQ, and JL conceived of the study, participated in its design, interpreted the molecular results, and drafted the manuscript; YY carried out the qPCR; CJ, PC, and YL collected the samples and clinical data; YQ and MC carried out the CMA, gap-PCR, and ddPCR; HP helped to revise the manuscript. All authors have read and approved the final manuscript.

## Funding

The work was supported by the National Key Research and Development Program of China (2018YFC1004900, 2016YFC1000703), Key Research and Development Program of Zhejiang Province (2019C03025), Zhejiang University—Lishui Cooperation Project (2018zdhz08), the National Natural Science Foundation of China (81300532, 81801441), and Zhejiang Key Laboratory of Organ Development and Regeneration (ZX15001017014).

## Conflict of Interest Statement

The authors declare that the research was conducted in the absence of any commercial or financial relationships that could be construed as a potential conflict of interest.

## Supplementary Material

Figure S1 CMA analysis of the proband, his sister, and their father and mother. The red arrows refer to the deletion. The black arrows refer to the normal copy in the deletion region.

Figure S2 Gap-PCR of the core family members using primer set (Gap2500-F/R). Lanes 1–5: Marker, II2, II1, III1, III2. Notice that II2 has one band about 10 kb in size, while the other three members have another band about 7 kb in size.

Figure S3 Gap-PCR of the father (II2) revealed heterozygous deletion in peripheral blood-derived and semen-derived DNA using primer set (4334-F/R). Lanes 1–3: Marker, blood, sperm. Notice that lane 3 has a lower band about 1.3 kb in size as lane 2. The PCR condition was as the same with that described in “MATERIALS AND METHODS” except the amplification cycles was changed to 34 cycles to obtain more PCR products for sequencing.
