# Supplementary figures and images for: Paternal Low-Level Mosaicism-Caused SATB2-Associated Syndrome
Source: Front Genet. 2019 Jul 2;10:630. doi: 10.3389/fgene.2019.00630 (PMC6614923; doi:10.3389/fgene.2019.00630)

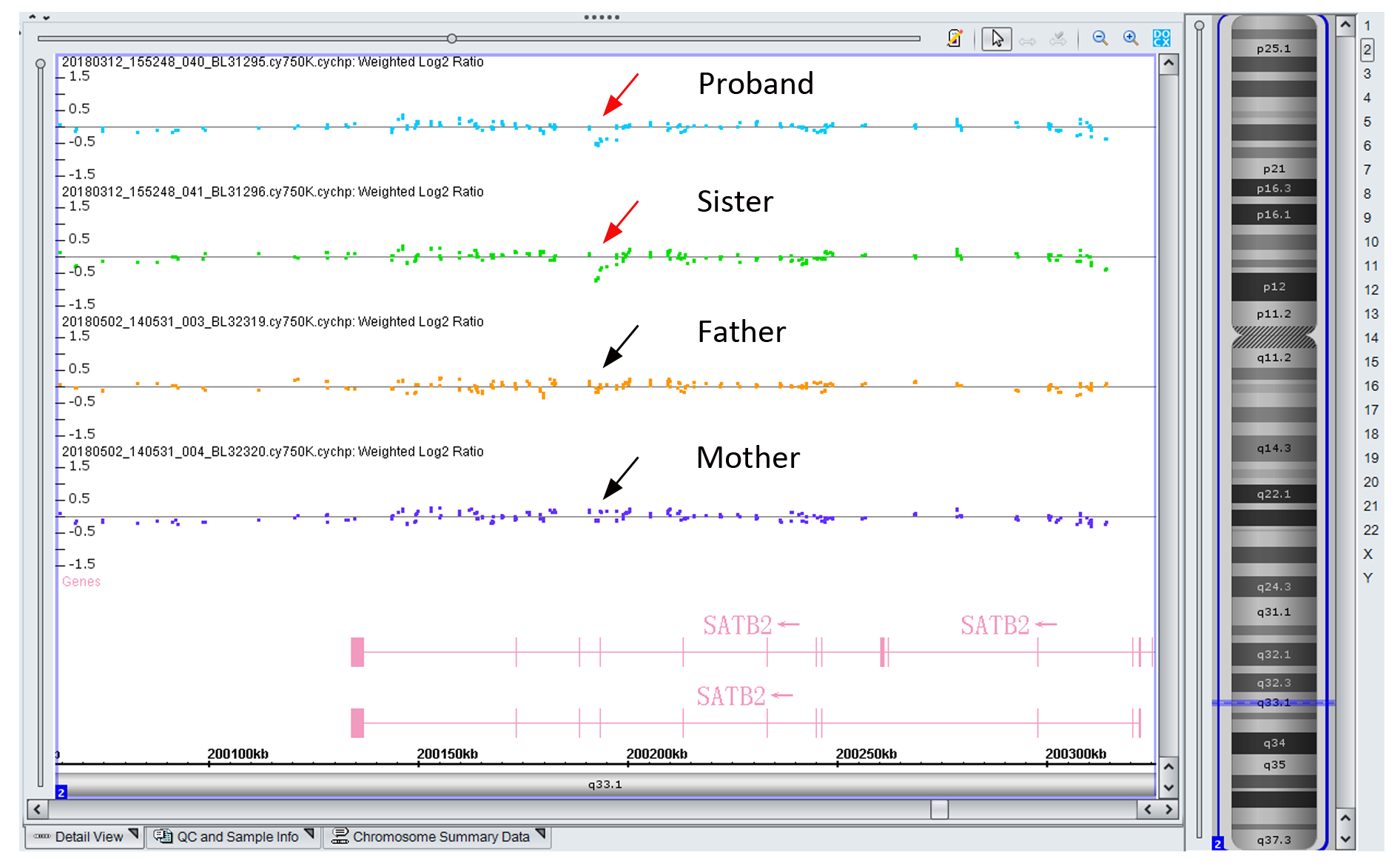

Supplement: Figure S1 — CMA analysis of the proband, his sister, and their father and mother. The red arrows refer to the deletion. The black arrows refer to the normal copy in the deletion region. [file Image_1.tif]

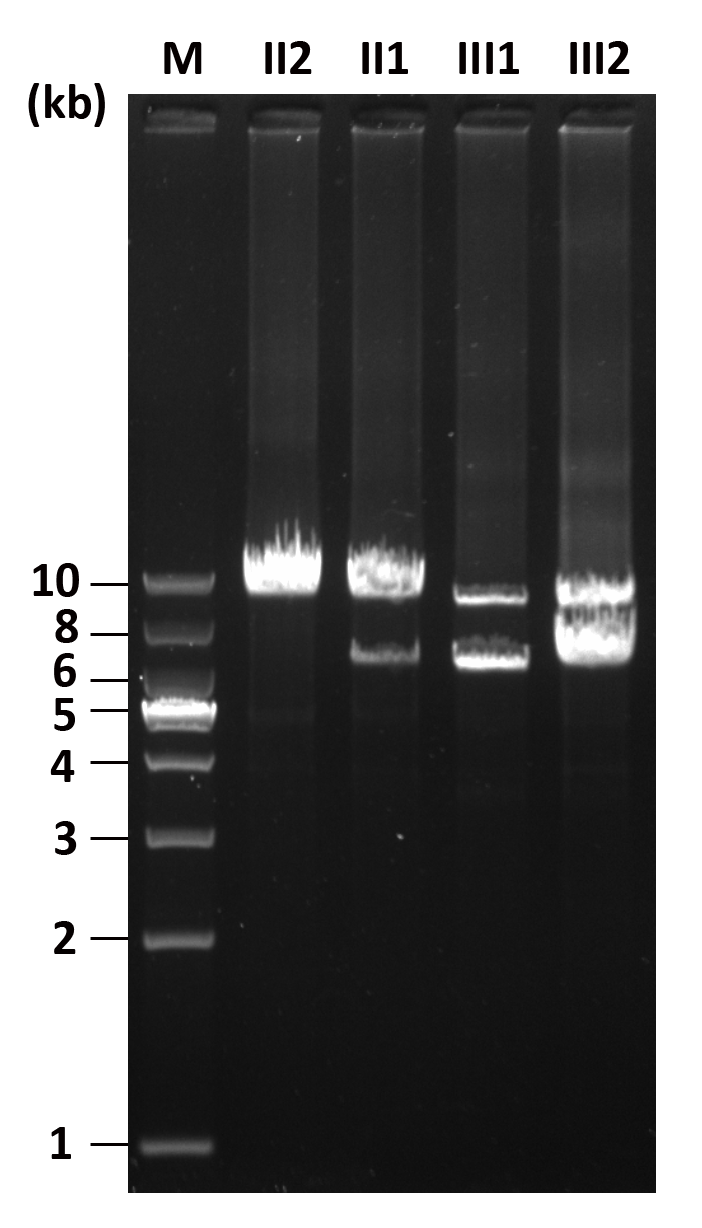

Supplement: Figure S2 — Gap-PCR of the core family members using primer set (Gap2500-F/R). Lanes 1–5: Marker, II2, II1, III1, III2. Notice that II2 has one band about 10 kb in size, while the other three members have another band about 7 kb in size. [file Image_2.tif]

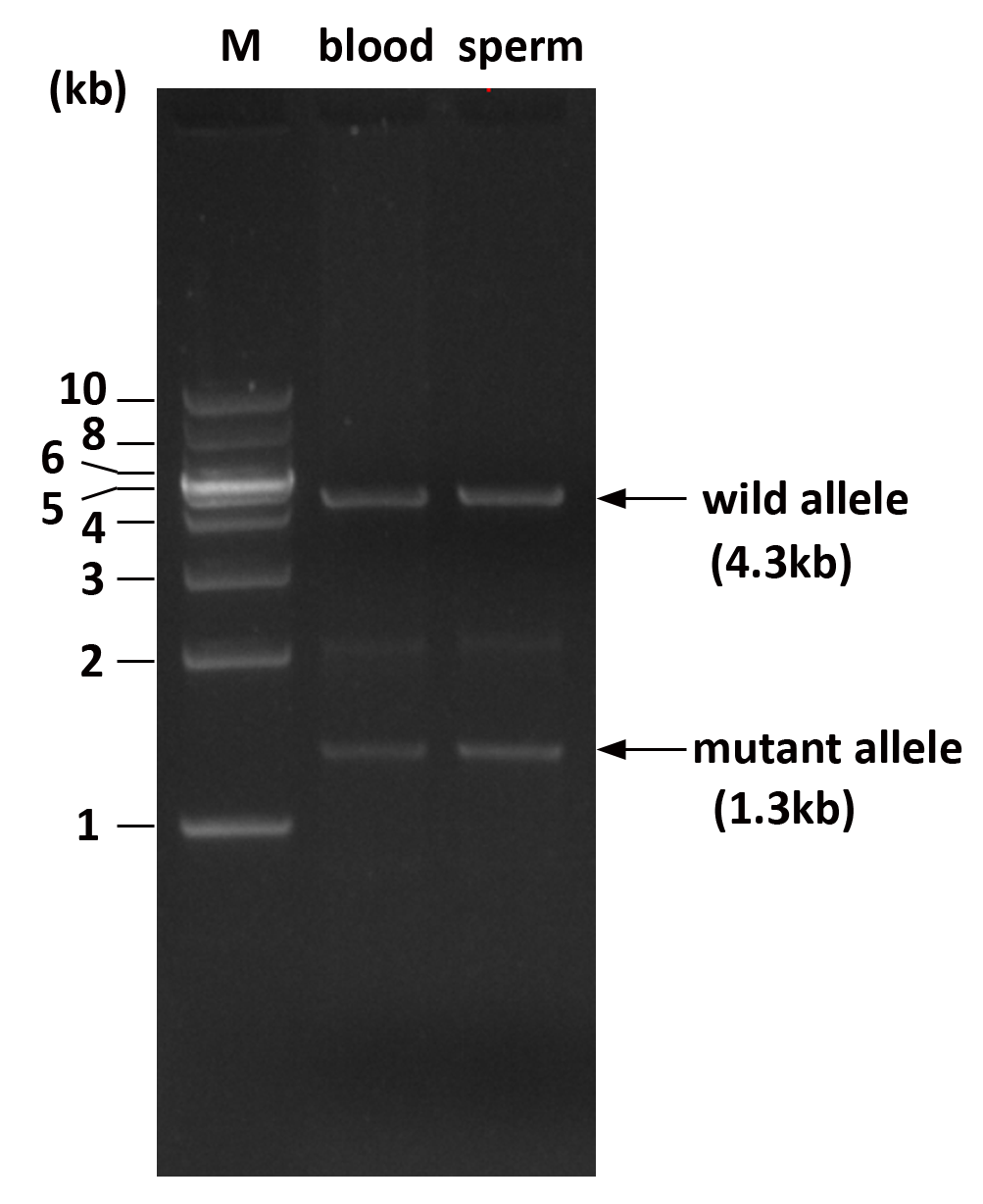

Supplement: Figure S3 — Gap-PCR of the father (II2) revealed heterozygous deletion in peripheral blood-derived and semen-derived DNA using primer set (4334-F/R). Lanes 1–3: Marker, blood, sperm. Notice that lane 3 has a lower band about 1.3 kb in size as lane 2. The PCR condition was as the same with that described in “MATERIALS AND METHODS” except the amplification cycles was changed to 34 cycles to obtain more PCR products for sequencing. [file Image_3.tif]
